# The Power of Microbiome Studies: Some Considerations on Which Alpha and Beta Metrics to Use and How to Report Results

**DOI:** 10.3389/fmicb.2021.796025

**Published:** 2022-03-03

**Authors:** Jannigje Gerdien Kers, Edoardo Saccenti

**Affiliations:** ^1^Laboratory of Microbiology, Wageningen University & Research, Wageningen, Netherlands; ^2^Laboratory of Systems and Synthetic Biology, Wageningen University & Research, Wageningen, Netherlands

**Keywords:** microbiota, power analysis, multivariate analysis, microbiome, sample size

## Abstract

**Background:**

Since sequencing techniques have become less expensive, larger sample sizes are applicable for microbiota studies. The aim of this study is to show how, and to what extent, different diversity metrics and different compositions of the microbiota influence the needed sample size to observe dissimilar groups. Empirical 16S rRNA amplicon sequence data obtained from animal experiments, observational human data, and simulated data were used to perform retrospective power calculations. A wide variation of alpha diversity and beta diversity metrics were used to compare the different microbiota datasets and the effect on the sample size.

**Results:**

Our data showed that beta diversity metrics are the most sensitive to observe differences as compared with alpha diversity metrics. The structure of the data influenced which alpha metrics are the most sensitive. Regarding beta diversity, the Bray–Curtis metric is in general the most sensitive to observe differences between groups, resulting in lower sample size and potential publication bias.

**Conclusion:**

We recommend performing power calculations and to use multiple diversity metrics as an outcome measure. To improve microbiota studies, awareness needs to be raised on the sensitivity and bias for microbiota research outcomes created by the used metrics rather than biological differences. We have seen that different alpha and beta diversity metrics lead to different study power: because of this, one could be naturally tempted to try all possible metrics until one or more are found that give a statistically significant test result, i.e., *p*-value < α. This way of proceeding is one of the many forms of the so-called *p*-value hacking. To this end, in our opinion, the only way to protect ourselves from (the temptation of) *p*-hacking would be to *publish* a statistical plan before experiments are initiated, describing the outcomes of interest and the corresponding statistical analyses to be performed.

## Introduction

For a few decades now, researchers have left culture-based methods and used molecular technologies, and more recently mostly sequencing-based approaches, to characterize microbial communities within a certain environment, referred to as the microbiome. In humans and animals, the microbiome has an important role in health and disease. For example, animals raised without or fewer microbes showed an underdeveloped immune system and are more susceptible to diseases (Inman et al., 2010; Mulder et al., 2011; Williams, 2014). Microbiome studies have as goal to investigate, characterize, and understand the compositional and functional variability of microbiomes. The question “What is different between different groups of interest?” can be translated into a hypothesis-testing procedure.

Hypothesis testing rests on the definition and choice of four parameters: (*i*) the effect size, i.e., the quantification of the outcome of interest (in the simple case, the difference between two groups); (*ii*) the sample size *n*, i.e., the number of samples (to be) collected; (*iii*) the power of tests 1 - β, i.e., the probability of the test of rejecting the null hypothesis when actually false; and (*iv*) the confidence level α, i.e., the probability of rejecting the null hypothesis when actually true.

It is necessary to perform power analysis before performing experiments. This is well acknowledged in all fields of research; however, microbiome studies are challenged with conflicting results (Knight et al., 2018). Underpowering and the failure to correct for false positives are among the causes underlying the lack of reproducibility of many biological findings (Begley and Ioannidis, 2015; Casals-Pascual et al., 2020).

The power of a test is linked to the probability β of accepting the null hypothesis when actually false (false-negative error or Type II error), and α describes the false-positive error or Type I error. Once acceptable error rates α (usually 0.05 or 0.01) and β (usually 0.2, although context-dependent) and the effect that one is interested to assess statistically are chosen, it is possible, at least in principle, to determine the optimal sample size, i.e., the number of samples that one needs to collect/analyze to obtain, with probability 1 −β, a statistically significant result with confidence α.

**FIGURE 1:**
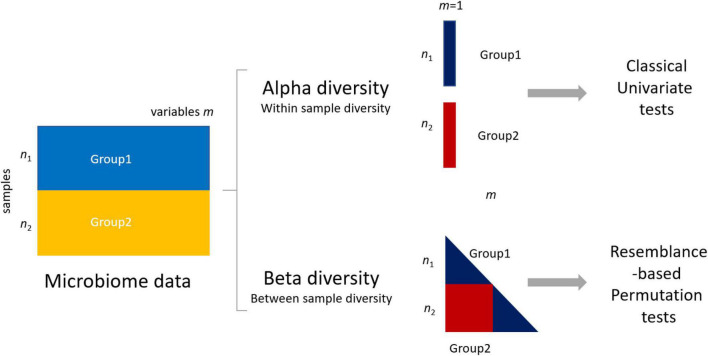
Differences between group 1 (data matrix **X**_1_ − n_1_ samples × *m*_1_ variables/microbial features) and group 2 in microbiome data can be assessed using either alpha (within-sample diversity) or beta (between-sample diversity) metrics. The use of alpha metrics allows the use of classical univariate testing, either parametric or nonparametric. The use of beta metrics leads to the use of permutation-based testing approaches like permutational multivariate ANOVA (PERMANOVA) (see section “Materials and Methods”).

Given the nature of microbiome data, it is possible to quantify differences between groups at two levels: the alpha (within-sample) and beta (between-sample) diversity (Figure 1). Alpha diversity metrics summarize the structure of a microbial community with respect to its richness (number of taxonomic groups), evenness (distribution of abundances of the groups), or both (Willis, 2019). Commonly used alpha metrics are phylogenetic diversity (PD) (Faith, 2006), observed number of amplicon sequence variants (ASVs) (Callahan et al., 2017), Chao1 (Chao, 1984), Simpson’s (Simpson, 1949; Lemos et al., 2011), and Shannon’s indices (Lemos et al., 2011; Magurran, 2013). Beta diversity metrics summarize which samples differ from one another by considering sequence abundances or considering only the presence–absence of sequences. Commonly used beta metrics are the Bray–Curtis (BC) dissimilarity (Bray and Curtis, 1957), Jaccard (Jaccard, 1912), unweighted UniFrac (UF) (Lozupone and Knight, 2005), and weighted UniFrac (Lozupone et al., 2007).

The choice of the diversity metrics affects the subsequent statistical testing and, as a result, how, and to which extent, power analysis can be performed.

With the use of an alpha diversity metric, a single diversity value is obtained for each sample containing measurements of *m* taxa; thus, the problem of assessing differences between two (or more) groups can be addressed with a univariate test, like *t-*test, ANOVA, or a nonparametric test. The use of a beta diversity metric implies that *all* samples are to be considered simultaneously, and several methods to compare groups of samples measured on *m* > 1 have been proposed like analysis of similarities (ANOSIM) (Clarke, 1993) and permutational multivariate ANOVA (PERMANOVA) (Anderson, 2001) to replace classical multivariate tests like the Hotelling *T*^2^ or multivariate ANOVA, which are in general not applicable. This happens because basic assumptions are not met, such as independence of the sample units, the multivariate normality of errors, homogeneity of variance–covariance matrices among the groups, or because the number of variables is larger than the number of samples, making it impossible to apply the test (Hanson and Weinstock, 2016; Gloor et al., 2017; Li et al., 2017; Weiss et al., 2017; Casals-Pascual et al., 2020).

While sample size and Type I and Type II errors are well-defined concepts, the definition of effect size depends on the outcome quantity of interest and how this quantity is mathematically defined. A fundamental step when performing power analysis is then to define the effect size: for a simple two-sample *t*-test, the effect size can be expressed as Cohen’s δ (Cohen, 2013).


(1) $$\delta =\frac{\left|\mu_{1}-\mu_{2}\right|}{\sigma }$$

where μ_1_ and μ_2_ are the population means of the two groups to be compared and σ^2^ is the pooled variance. Since μ_1_ and μ_2_ are population parameters that are inaccessible and on which we want to perform inference, an *a priori* estimation, or educated guess, is necessary. This can be accomplished by taking the sample means *m*_1_ and *m*_2_ and pooled variance *s*^2^ from a pilot study or existing data to obtain estimates of the population parameters. A critical aspect that is not sufficiently acknowledged is that the effect size from Equation (1) is sensitive to the particular diversity metric used to the point that sample size calculations can be severely affected.

The aim of this study is to show how, and to what extent, different diversity metrics influence the sample size needed to assess the statistical significance of dissimilarities between different microbial communities. Both simulated and empirical 16S rRNA gene amplicon sequence datasets are used to perform retrospective calculations of the empirical power for microbiota studies. A broad selection of alpha and beta diversity metrics was used to compare the different microbiota datasets.

This study generated insight into the sensitivity and bias of certain statistical methods used in microbial ecology on microbiota research outcomes. We conclude with some recommendations for the reporting of power analysis and sample size calculations for microbiome studies.

## Materials and Methods

### Literature Search

To support the choice of alpha and beta diversity metrics to consider in our comparison, we performed a literature search on PubMed^1^ with a query: (microbiota [Title] OR microbiome [Title]) NOT Review [Publication Type] 2020/01:2020/02 [Date of Publication]). This strategy aimed to include a broad scope of microbiota studies. We limited our search to studies published in English with free full text.

### Alpha Diversity Metrics

#### Richness

Richness is the number of taxa, most often defined as an operational taxonomic unit (OTU) or ASVs observed (Callahan et al., 2017), where *s* is the number of observed taxa, calculated as (Colwell, 2009)


(2) $$S_{rich}=\sum_{s>0}1_{s}$$

#### Phylogenetic Diversity

PD is a phylogenetically weighted measure of richness. Although the name suggests diversity, it does not take into account the abundance of taxa. The PD is defined as the sum of the lengths of all those branches on the tree that span the members of the set, given the phylogenetic tree spanning *s* taxa (Faith, 2006):


(3) $$PD=\sum_{i}b_{i},$$

where *s* is the number of observed taxa and *b*_*i*_ is the length of the *i*th branch in the tree. Index *i* runs on all branches.

#### Chao1

The Chao1 index is an abundance-based nonparametric estimator of taxa richness (Chao, 1984). It is defined as


(4) $$Chao1=s+\frac{F_{1}\left(F_{1}-1\right)}{2\left(F_{2}-1\right)},$$

where *s* is the number of observed taxa, and F_1_ and F_2_ are the number of OTU/ASV with only one sequence (i.e., “singletons”) and two sequences (i.e., “doubletons”). This metric assumes the number of organisms identified for a taxon to follow a Poisson distribution. The definition rests on the concept that rare taxa bring most information about the number of missing taxa. This index gives more weight to the low-abundance taxa, and only the singletons and doubletons are used to estimate the number of missing taxa (Chao, 1984). This index is particularly useful for datasets skewed toward low-abundance taxa (Hughes et al., 2001; Kim et al., 2017). However, singletons and doubletons are often removed from 16S rRNA amplicon sequence datasets because of the difficulty in robustly differentiating singleton errors from real singleton sequences (Allen et al., 2016; Callahan et al., 2016).

#### Shannon’s Index

Shannon’s index *H* is an estimator of taxa diversity, combining richness and evenness) (Lemos et al., 2011; Magurran, 2013). It is defined as


(5) $$H=-\sum_{i=1}^{s}p_{i}\text{log}\left(p_{i}\right),$$

where *s* is the number of OTU/ASV and p_*i*_ is the proportion of the community represented by the *i*th OTU/ASV. Basically, this index is the entropy associated with a given sample and quantifies the uncertainty in predicting the taxa identity of an individual selected at random from the sample. Shannon’s index uses the relative abundances of different taxa; thus, diversity depends on both taxa richness and evenness with which organisms are distributed among the different taxa. This index places a greater weight on taxa richness (Kim et al., 2017).

#### Simpson’s Index

Simpson’s index *D* is an estimator of taxa diversity, combining richness and evenness (Simpson, 1949; Lemos et al., 2011). It is defined as


(6) $$D=\frac{1}{\sum_{i=1}^{s}p_{i}^{2}},$$

where *s* is the number of OTU/ASV and p_*i*_ is the proportion of the community represented by the *i*th OTU/ASV. This index considers taxa evenness more than taxa richness in its measurement (Kim et al., 2017); it indicates the taxa dominance and gives the probability of two individuals that belong to the same taxa being randomly chosen. It varies from 0 to 1, and the index increases as the diversity decreases (Kim et al., 2017).

### Metrics for Beta Diversity

#### Bray–Curtis Dissimilarity

The BC index (Bray and Curtis, 1957) measures the compositional dissimilarity between the microbial communities of two samples *i* and *j* based on counts on each sample. It is defined as


(7) $$BC=1-\frac{2C_{ij}}{S_{i}+S_{j}},$$

where *C*_*ij*_ is the sum of the smallest values for only those taxa in common between the sample *i* and *j*, and *S*_*i*_ and *S*_*j*_ are the total number of taxa counted in sample *i* and *j*, respectively. This index ranges between 0 (the two samples share all taxa) and 1 (the two samples do not share any taxa). It gives more weight to common taxa (Borcard et al., 2018). The BC dissimilarity is computed pairwise between all samples.

#### Jaccard Distance

The Jaccard distance *J* between two samples *i* and *j* is defined as *J* = 1 - *J*(*i*, *j*), where *J*(*i*, *j*) is the Jaccard index, which is defined as


(8) $$J\left(i,j\right)=\frac{\left|i\cap j\right|}{\left|i\cup j\right|},$$

which is the ratio between the number of members that are common between the two samples and the number of members that are distinct; it is a measure of similarity for the two communities and ranges between 0 (the communities are different) and 1 (the two communities are identical).

#### UniFrac Distances

UF and weighted UniFrac distances between two samples take into account the phylogenetic tree and thus phylogenetic distances between community members (Lozupone et al., 2007). In UF, the distance is calculated as the fraction of the branch length, and in weighted UniFrac, branch lengths are weighted by the relative abundance of sequences. The sum of unshared branch lengths is divided by the sum of all tree branch lengths, which results in the fraction of total unshared branch lengths that is defined as


(9) $$\sum_{i}^{n}b_{i}\times \left[\frac{A_{i}}{A_{T}}-\frac{B_{i}}{B_{T}}\right].$$

Lozupone et al. (2007) defined n as the total number of branches in the tree; *b*_*i*_ as the length of branch *i*; *A*_*i*_ and *B*_*i*_ as the numbers of sequences that descend from branch *i* in communities *A* and *B*, respectively; and *A*_*T*_ and *B*_*T*_ as the total numbers of sequences in communities A and *B*, respectively. In order to control for unequal sampling effort, *A*_*i*_ and *B*_*i*_ are divided by *A*_*T*_ and *B*_*T*_ (Lozupone et al., 2007).

### Experimental Datasets

#### Chickdata Dataset

This dataset contains 16S rRNA gene amplicon sequence data obtained from a broiler chicken experiment. The dataset is described in detail in Kers et al. (2019); briefly, chickens were raised under three different housing conditions with the same medium-chain fatty acid (MCFA) feed intervention. Between those housing conditions, bird management was kept as similar as possible. At the hatchery, the chicks were randomly allocated to three different experimental facilities. Dataset A contains 70 broilers from a grow-out feed trial facility, dataset B contains 70 broilers raised at a floor stable, and dataset C contains 70 broilers raised in isolators. A feed intervention was used as a tool to create differences in cecal microbiota between broilers within the same housing condition.

#### Human Microbiome Project Dataset

This dataset was obtained from the Human Microbiome Project (HMP) phase I (Huttenhower et al., 2012). It contains 16S rRNA gene amplicon sequence data of 169 stool samples, 150 oral samples, 86 vaginal samples, and 69 skin samples. The bodyside microbiomes are all diverse in terms of community membership (Huttenhower et al., 2012).

In all datasets, ASVs were defined as unique sequences. All data were analyzed using NG-Tax (Ramiro-Garcia et al., 2016). Taxonomy was assigned using the SILVA 128 16S rRNA gene reference (Quast et al., 2013). An overview of dataset characteristics (sample size, the number of ASVs, and mean values of different alpha and beta diversity metrics) is shown in Table 1.

**TABLE 1:** Overview of dataset characteristics of the different datasets.

A	*N*	ASV	PD	Shannon	Chao1	Simpson
Chickdata A Feed1	35	780	28.3 (2.9)	4.1 (0.3)	136.3 (20.8)	0.96 (0.02)
Chickdata A Feed2	35	794	28.2 (2.8)	4.1 (0.3)	137.9 (22.0)	0.96 (0.02)
Chickdata B Feed1	35	537	22.6 (3.7)	3.5 (0.5)	109.6 (21.3)	0.90 (0.08)
Chickdata B Feed2	35	588	26.9 (2.7)	4.0 (0.3)	139.5 (20.2)	0.95 (0.02)
Chickdata C Feed1	35	466	17.4 (3.1)	3.2 (0.5)	79.0 (17.7)	0.90 (0.06)
Chickdata C Feed2	35	518	20.2 (2.3)	3.7 (0.3)	98.1 (14.4)	0.95 (0.02)
HMP gut	168	1,996	17.3 (3.7)	3.2 (0.6)	70.1 (21.7)	0.9 (0.1)
HMP oral	150	1,740	22.6 (4.6)	3.4 (0.5)	82.6 (21.2)	0.9 (0.1)
HMP skin	69	899	12.2 (6.3)	1.7 (0.6)	41.4 (20.7)	0.7 (0.1)
HMP vaginal	86	678	8.6 (3.7)	1.1 (0.6)	31.8 (10.2)	0.4 (0.2)

**B**	***N***	**ASV**	**BC**	**Jaccard**	**UF**	**WUF**

Chickdata A Feed1	35	780	0.74 (0.09)	0.84 (0.06)	0.40 (0.05)	0.30 (0.08)
Chickdata A Feed2	35	794	0.71 (0.09)	0.83 (0.06)	0.39 (0.05)	0.30 (0.07)
Chickdata B Feed1	35	537	0.59 (0.13)	0.74 (0.10)	0.41 (0.12)	0.27 (0.10)
Chickdata B Feed2	35	588	0.63 (0.12)	0.77 (0.09)	0.35 (0.06)	0.28 (0.09)
Chickdata C Feed1	35	466	0.72 (0.15)	0.83 (0.12)	0.45 (0.12)	0.33 (0.09)
Chickdata C Feed2	35	518	0.69 (0.11)	0.81 (0.08)	0.36 (0.07)	0.29 (0.06)
HMP gut	168	1,996	0.80 (0.10)	0.89 (0.07)	0.54 (0.08)	0.39 (0.13)
HMP oral	150	1,740	0.70 (0.13)	0.82 (0.09)	0.49 (0.11)	0.33 (0.12)
HMP skin	69	899	0.59 (0.20)	0.72 (0.16)	0.66 (0.10)	0.29 (0.17)
HMP vaginal	86	678	0.71 (0.29)	0.79 (0.24)	0.70 (0.11)	0.21 (0.15)

**C**	***n***	**ASV**	**BC**	**Jaccard**	**UF**	**WUF**

Chickdata A Feed1	35	780	0.51 (0.06)	0.59 (0.04)	0.28 (0.04)	0.21 (0.06)
Chickdata A Feed2	35	794	0.50 (0.05)	0.58 (0.04)	0.27 (0.03)	0.21 (0.05)
Chickdata B Feed1	35	537	0.41 (0.09)	0.51 (0.07)	0.28 (0.09)	0.19 (0.08)
Chickdata B Feed2	35	588	0.44 (0.07)	0.54 (0.05)	0.24 (0.04)	0.20 (0.06)
Chickdata C Feed1	35	466	0.51 (0.07)	0.58 (0.05)	0.32 (0.08)	0.24 (0.05)
Chickdata C Feed2	35	518	0.48 (0.07)	0.57 (0.05)	0.25 (0.05)	0.20 (0.04)
HMP gut	168	1,996	0.57 (0.07)	0.63 (0.04)	0.38 (0.06)	0.27 (0.09)
HMP oral	150	1,740	0.49 (0.09)	0.58 (0.06)	0.33 (0.09)	0.23 (0.08)
HMP skin	69	899	0.40 (0.15)	0.50 (0.12)	0.46 (0.06)	0.20 (0.13)
HMP vaginal	86	678	0.51 (0.23)	0.56 (0.18)	0.49 (0.05)	0.14 (0.12)

*The mean alpha and beta diversity, and associated standard deviation (between brackets) (A, B). The mean of the beta diversities is also calculated as the mean distance of groups members to the group centroid (C). N = sample size. Feed1 is the intervention without the same medium-chain fatty acid. PD, the phylogenetic diversity; ASV, amplicon sequence variant; HMP, Human Microbiome Project; BC, Bray–Curtis dissimilarity; UF, unweighted UniFrac; WUF, weighted UniFrac.*

### Simulated Datasets

We simulated two different scenarios described by Simulated datasets 1 and 2. An overview of the characteristics of the simulated datasets is shown in Table 2.

**TABLE 2:** Overview of dataset characteristics of the simulated datasets.

A	*N*	ASV	PD	Shannon	Observed/Chao1	Simpson
Simulation 1—2%	169	1,955	17.2 (3.7)	3.1 (0.6)	69.2 (21.4)	69.2 (0.1)
Simulation 1—5%	169	1,895	17.0 (3.6)	3.1 (0.6)	66.2 (20.8)	66.2 (0.1)
Simulation 1—10%	169	1,795	16.7 (3.6)	3.0 (0.6)	62.7 (19.8)	62.7 (0.1)
Simulation 1—25%	169	1,496	15.7 (3.4)	2.9 (0.5)	55.3 (17.0)	55.3 (0.1)
Simulation 1—50%	169	997	12.8 (2.8)	2.5 (0.5)	35.5 (10.7)	35.5 (0.1)
Simulation 1—75%	169	498	8.8 (2.2)	2.1 (0.4)	16.4 (6.1)	16.4 (0.1)
Simulation 2—1%	169	1,995	106.9 (1.2)	7.1 (0.0)	1,385.5 (19.9)	1,385.5 (0.0)
Simulation 2—2%	169	1,995	107.0 (1.2)	7.1 (0.0)	1,393.4 (19.2)	1,393.4 (0.0)
Simulation 2—5%	169	1,995	107.7 (1.1)	7.1 (0.0)	1,411.5 (18.3)	1,411.5 (0.0)
Simulation 2—10%	169	1,995	108.3 (1.2)	7.1 (0.0)	1,439.5 (19.6)	1,439.5 (0.0)
Simulation 2—15%	169	1,995	109.4 (1.1)	7.1 (0.0)	1,472.2 (19.4)	1,472.2 (0.0)
Simulation 2—20%	169	1,995	110.3 (1.2)	7.1 (0.0)	1,502.8 (17.0)	1,502.8 (0.0)

**B**	***N***	**ASV**	**BC**	**Jaccard**	**UF**	**WUF**

				0.89 (0.07)		
Simulation 1—2%	169	1,955	0.80 (0.10)	0.89 (0.07)	0.53 (0.08)	0.39 (0.14)
Simulation 1—5%	169	1,895	0.81 (0.10)	0.89 (0.07)	0.54 (0.08)	0.39 (0.14)
Simulation 1—10%	169	1,795	0.80 (0.10)	0.88 (0.07)	0.53 (0.08)	0.39 (0.14)
Simulation 1—25%	169	1,496	0.79 (0.11)	0.86 (0.07)	0.53 (0.08)	0.39 (0.14)
Simulation 1—50%	169	997	0.77 (0.13)	0.91 (0.09)	0.53 (0.08)	0.40 (0.15)
Simulation 1—75%	169	498	0.84 (0.12)	0.67 (0.08)	0.59 (0.10)	0.50 (0.16)
Simulation 2—1%	169	1,995	0.50 (0.01)	0.66 (0.01)	0.24 (0.01)	0.06 (0.01)
Simulation 2—2%	169	1,995	0.49 (0.01)	0.64 (0.01)	0.24 (0.01)	0.06 (0.01)
Simulation 2—5%	169	1,995	0.47 (0.01)	0.61 (0.01)	0.23 (0.01)	0.05 (0.01)
Simulation 2—10%	169	1,995	0.43 (0.01)	0.58 (0.01)	0.22 (0.01)	0.05 (0.00)
Simulation 2—15%	169	1,995	0.40 (0.01)	0.55 (0.01)	0.21 (0.01)	0.05 (0.00)
Simulation 2—20%	169	1,995	0.38 (0.01)		0.20 (0.01)	0.04 (0.01)

*The mean alpha and beta diversity, and associated standard deviation (between brackets) (A, B). N = sample size. PD, phylogenetic diversity; BC, Bray–Curtis dissimilarity; ASV, amplicon sequence variant; UF, unweighted UniFrac; WUF, weighted UniFrac.*

Simulated dataset 1 is built starting from 1,995 microbial features observed in 169 stool samples from the HMP, indicated as **X**_1_ in the following. Datasets (named **X**_2_ for the sake of simplicity) were created where 2, 5, 10, 25, 50, and 75% of bacterial features were randomly removed. This simulation generates data under an exist–non-exist binary scenario where bacterial features are either present or absent in **X**_1_ and **X**_2_.

Simulated dataset 2 is a case–control scenario (**X**_1_ controls and **X**_2_ cases, with 1,995 features and 169 samples each) where one-fourth of bacterial features in **X**_2_ are 1, 2, 5, 10, 15, and 20% more abundant than in **X**_1_. Both simulated datasets have the same phylogenetic structure as the HMP dataset. This simulation thus generates data under a differential abundant scenario where bacterial features are present in the different compositions in **X**_1_ and **X**_2_.

### Statistical Tests for Group Differences

#### Univariate Statistical Analysis

Differences between groups using alpha diversity as determined by using PD, richness (defined as observed), Chao1, Simpson, and Shannon were assessed using the Kruskal–Wallis test (Kruskal and Wallis, 1952). A significance threshold α = 0.01 was used in all calculations.

#### Permutational Multivariate ANOVA

The differences between groups using beta diversity as determined by using the BC, Jaccard, UF, and weighted UniFrac were assessed using the PERMANOVA (Anderson, 2001). PERMANOVA is a robust approach to compare groups of samples measured on *m* > 1 variables. It constructs ANOVA-like test statistics from a matrix of resemblances (distances, dissimilarities, or similarities) calculated among the sample units and assesses the significance of the observed differences using random permutations of observations among the groups (Anderson and Walsh, 2013). The null hypothesis H_0_ tested by PERMANOVA is that the centroids of the groups (in the space of the chosen resemblance measure) are the same for all groups. This test assumes that samples are exchangeable under the null hypothesis, are independent, and have similar multivariate dispersion. The PERMANOVA test statistic is a pseudo ANOVA *F*-ratio:


(10) $$F=\frac{SS_{B}\backslash \left(g-1\right)}{SS_{W}\backslash \left(n-g\right)}$$

where *SS*_*B*_ is the total sum of squares of the (diss)similarities between groups, *SS*_*W*_ is the total sum of squares of the (diss)similarities within groups, *g* is the number of groups, and *n* is the total number of samples.

The significance of the *F*-statistics is calculated by means of permutations (*k* = 9,999). The distribution of *F* under the null hypothesis is generated by permuting *g* times the sample group labels and recalculating *F* on the permuted data. Significance is expressed as a *p*-value calculated as the fraction of permuted *F*-statistics, which are equal to or greater than the pseudo *F*-ratio observed on the original data.

### Data Subsampling

The experimental and simulated data were used to generate *K* random datasets of different sizes to take into account both data generation variability and the calculation of the empirical power. More specifically, from *N*_1_ × *m* and *N*_2_ × *m* data matrices **X**_1_ and **X**_2_ (either experimental or from Simulations 1 and 2), we randomly sampled with replacements *K* = 1,000 *n*_1_ × *m* and *n*_2_ × *m* datasets for different sample sizes *n*_1_ and *n*_2_. For the sake of simplicity, we consider *n*_1_ = *n*_2_ = *n*, and we varied *n* between 5 and 50 or 100 in steps of 5. We used *K* = 1,000 for the analysis of univariate analysis (alpha) and *K* = 100 for the analysis of multivariate analysis (beta), both simulated and experimental data. In total, more than 40,000 randomly generated datasets were analyzed.

### Calculation of the Empirical Power

A statistical test to assess the difference between **X**_1_ and **X**_2_ (as quantified by any of the alpha and beta metrics) at significance level α = 0.01 under the assumption of the null hypothesis being false was applied on the *K* randomly generated datasets for different sample sizes *n*. The empirical power of the test is defined as the empirical probability *EPr* of H_0_ being rejected, calculated as


(11) $$EPr=\frac{\#\left(H_{0}rejected|H_{0}false\right)}{K}$$

where #() indicates the number of times that H_0_ is rejected.

### Software

All statistical analyses were performed in R version 4.0.2 (R Foundation for Statistical Computing, Austria; R Core Team, 2008), using the following packages: Phyloseq, Microbiome, and Vegan (Oksanen et al., 2020; McMurdie and Holmes, 2013; Lahti and Shetty, 2017). PERMANOVA was performed using the *adonis* function for the Vegan package. Other power calculations were performed using the G*power software (Faul et al., 2007) using the “Means: Difference between two independent means (two groups)” as Statistical test and “*a priori*” and “*post hoc*” option for the Type of Power analysis. Differentially abundant microbiota profiles were simulated with the microbiomeDASim R package (Williams et al., 2019) using the *gen_norm_microbiome* function. The R scripts can be found on the Github page: https://github.com/mibwurrepo/KersSaccenti-Power.

## Results

### Motivation Example

We begin with a motivational example to show how the choice of the diversity metrics affects the power of a microbiome study and how the same study may be underpowered if a different metric is used.

Let us suppose we want to plan an experiment to assess whether gut and oral microbial communities are different. A very simple and basic study design would be to collect *n*_1_ = *n*_2_ gut and oral samples and compare the alpha diversity between the two conditions (gut vs. oral) using a two-sample Kruskal–Wallis *t*-test.

**FIGURE 2:**
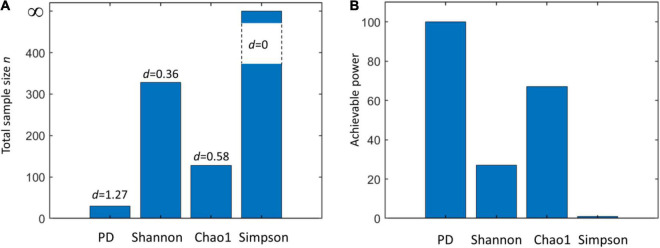
**(A)** Total sample size (*n*) required to assess the statistical significance of a given effect *d* using a two-sample Kruskal–Wallis test with a power equal to 0.8 and confidence α = 0.01 when using different alpha metrics (phylogenetic diversity (PD), Shannon’s, Chao1, and Simpson’s indices). **(B)** Achievable power attainable by a Kruskal–Wallis test using a total sample size *n* = *n*_1_+ *n*_2_ = 50 + 50 = 100 using different alpha metrics (same as **A**). Note that for a null effect (*d* = 0), the achievable power coincides with α. Effect sizes are calculated from the Human Microbiome Project (HMP) data (comparison of skin and vaginal microbiome) reported in Table 1.

We can base our estimation *d* of a very similar effect size δ on data from HMP (Table 1). Using four different alpha metrics and Equation (1), we obtained *d* = 1.27 (PD), *d* = 0.3621 (Shannon), *d* = 0.58 (Chao1), and *d* = 0 (Simpson). These values are markedly different: fixing the power to 0.8 (β = 0.2) and confidence α = 0.05 will lead to dramatically different required total sample size (Figure 2A). This clearly indicates that microbiome studies may be severely underpowered depending on which alpha metric was used to compare two (or more) groups.

We also explored the achievable power by fixing the sample size (*n* = *n*_1_ + *n*_2_ = 50 + 50 = 100) and using different effect sizes (Figure 2B). Consistently with what is observed in Figure 2A, results vary strongly, providing a clear indication of the risk of underpowering when Shannon’s diversity is used.

Note that with the use of beta diversity metrics, performing *a priori* power analysis becomes much more complicated. The classical tools for power analysis cannot be applied since the statistical tools are not parametric: solutions have been proposed in the literature; see, for instance (La Rosa et al., 2012; Kelly et al., 2015; Xia et al., 2018).

### Literature Search

Our literature search returned 632 papers matching the search criteria. We selected randomly 100 papers, and of those, the materials and methods or full text were investigated to obtain an overview of the most frequently used alpha and beta diversity metrics and the sample sizes used. Of the 100 full-text papers, 92% of the papers contained alpha metrics, and 83% of the papers contained beta metrics.

In 58% of the papers, more than one alpha metric was used. In 21% of the papers, more than one beta metric was used. An overview of the frequency of the different metrics showed that Shannon’s index and the BC dissimilarity are the most common metrics (Table 3). There was a wide variance in the used sample size, defined as the smallest number per group: 46% of the papers had a sample size of ≤ 10 samples, 34% of the papers used between 11 and 50 samples, 7% of the papers used between 51 and 100 samples, 10% of the papers used between 101 and 1,000 samples, and three papers used > 1,000 samples.

**TABLE 3:** The frequency of the different alpha and beta metrics in published papers with microbiome or microbiota in the title and published between January 2020 and February 2020 (*n* = 100, multiple metrics per paper were often used).

Alpha metrics	n	Beta metrics	n
Shannon index	78	Bray–Curtis	41
Chao1	39	Weighted UniFrac	35
Observed OTU/ASV	32	Unweighted UniFrac	21
(Inverse) Simpson	29	Jaccard	4
Phylogenetic	7	Euclidean	3
ACE	5	Jackknifed	2
Coverage	3	Yue and Clayton	2
Pielou	3	Sorensen	1
Sobs	2	Jensen–Shannon	1
Gini–Simpson	1		
Shannon–Wiener	1		

*OTU, operational taxonomic unit; ASV, amplicon sequence variant.*

This (small) literature offers an indication of what the most used alpha and beta metrics are for the analysis of microbiome data. The results are not surprising and agree with the author’s experience. We can note that none of the papers screened mentioned the use of Hill’s numbers (Hill, 1973), a mathematically unified family of diversity indices (differing among themselves only by a parameter) that incorporate species richness and species relative abundances (Chao et al., 2016). The use of Hill’s number has found consensus in ecology (Jost, 2007; Ellison, 2010), and they have also been used for the analysis of microbiome data (Haegeman et al., 2013; Ma, 2018; Ma and Li, 2018). However, their use seems to be not widespread, and their utility is not fully acknowledged: in this study, we will focus on the more commonly used metrics.

### The Power of Microbiome Studies

As shown in the motivational example, the choice of a particular alpha (and beta) diversity metric determines the number of samples required to achieve a predetermined power. Based on this observation, we examined two simulated datasets using both alpha and beta diversity metrics to understand the relationship between the sample size, the observed power, and the diversity metrics, together with two experimental datasets (chicken and HMP datasets).

As representatives of testing procedures using alpha and beta diversity measures to compare two groups, the Kruskal–Wallis test (for alpha metrics) and PERMANOVA (for beta metrics), respectively, were selected. The Kruskal–Wallis test is the nonparametric choice for comparing two groups when the normality assumption does not hold. When comparing two (or more) groups using beta diversity metrics, PERMANOVA and ANOSIM (Clarke, 1993) are popular choices. The two approaches are equally popular (359 hits on PubMed for PERMANOVA and 341 for ANOSIM); however, Anderson and Walsh (2013) showed that while both approaches are sensitive to unbalanced designs and differences in variance within groups, PERMANOVA is a more robust approach: on this ground, we based our choice for PERMANOVA.

While in this study we focus only on inferential approaches for the analysis of microbiome data, as carried on using univariate and multivariate tests, we should comment that sample size is also relevant for exploratory approaches like principal component analysis (PCA), principal coordinate analysis (PCoA), and multidimensional scaling (MDS). These approaches are not inferential (as long one does not consider the inferential setting for dimension assessment; Saccenti and Timmerman, 2016, 2017), but the number of samples affects the reliability and stability of loadings and stability; thus, asking what the (minimal) sample size is to obtain stable and reproducible component loading estimations is relevant. Very little is known on the topic in classical PCA (Saccenti and Timmerman, 2016) and factor analysis setting (MacCallum et al., 1999) from a theoretical point of view, which cannot be directly extended to PCoA and MDS, which are the most commonly used approaches in microbiome analysis.

### Power Analysis of Simulated Datasets

**FIGURE 3:**
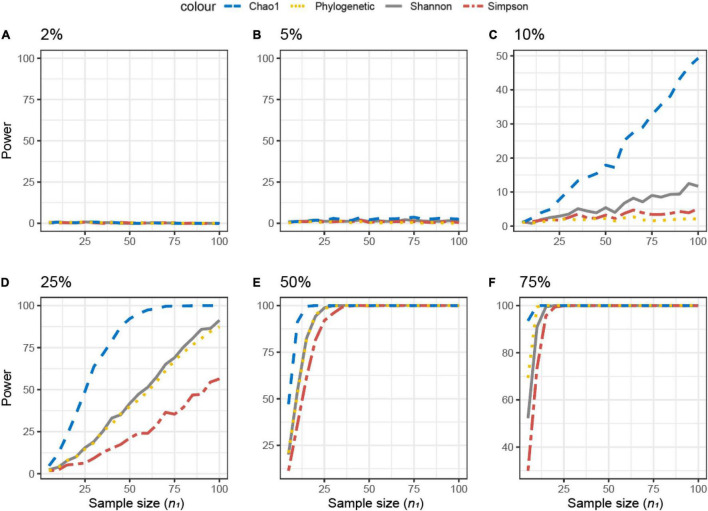
Empirical power of a two-sample Kruskal–Wallis test for the univariate comparison of two simulated microbiome datasets **X**_1_ and **X**_2_ [Simulated dataset 1: this simulation generates data under an exist–non-exist binary scenario where bacterial features are either present or absent in **X**_1_ and **X**_2_. The data have the same phylogenetic structure of the Human Microbiome Project (HMP) Gut data described in Table 1] using different alpha diversity metrics (phylogenetic, Chao1, Shannon’s, and Simpson’s indices; see Equations 3–6 in section “Materials and Methods”). The empirical power is calculated using Equation (11) as a function of the sample size *n*_1_ of group 1 (with *n*_2_ = *n*_1_ and total sample size *n* = *n*_1_ + *n*_2_) using *K* = 1,000 replications. **(A–F)** The analysis of data simulations in which 2, 5, 10, 25, 50, and 75% of bacterial features are not present in dataset **X**_2_ with respect to **X**_1_, respectively.

For the simulated datasets, the effect size is known *a priori*, and it is expressed as the % of differentially abundant or present/absent microbial features (ASV) (Figure 3). The achievable power for Simulated dataset 1 is shown as a function of the sample size (*n*) for different percentages of present/absent ASV. If 2%, 5% of the ASVs are deleted from the dataset, none of the alpha diversity metrics was able to capture the difference between datasets **X**_1_ and **X**_2_, irrespective of the sample size used (Figures 3A,B). When more than 10% of ASVs were removed from dataset **X**_2_ (Figures 3C–E), all measures were somehow able to capture the difference, but the resulting actual power was very different. Overall, Chao1 and observed diversity allowed higher power with the lower sample size (are more sensitive to observe differences), especially in the medium range of differences (10–25%, Figures 3C,D), whereas differences are minimal for > 25%. Note that in contrast with the motivation example, here, the PD was not the metric resulting in the smallest sample size.

**FIGURE 4:**
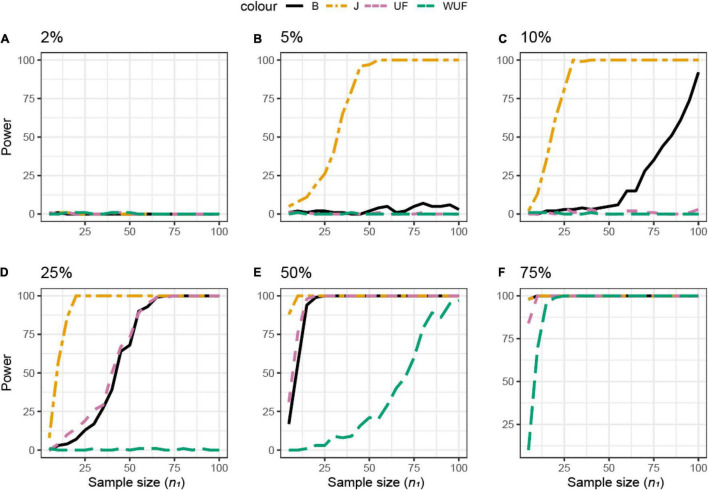
Empirical power of a permutational multivariate ANOVA (PERMANOVA) test for the multivariate comparison of two simulated microbiome datasets **X**_1_ and **X**_2_ [Simulated dataset 1: this simulation generates data under an exist–non-exist binary scenario where bacterial features are either present or absent in **X**_1_ and **X**_2_. The data have the same phylogenetic structure of the Human Microbiome Project (HMP) Gut data described in Table 1] using different beta diversity metrics [Bray–Curtis dissimilarity **(B)**, Jaccard (J), unweighted UniFrac (UF), and weighted UniFrac (WUF) distances; see Equations (7)–(9), and (6) in section “Materials and Methods”]. The empirical power is calculated using Equation (11) as a function of the sample size *n*_1_ of group 1 (with *n*_2_ = *n*_1_ and total sample size *n* = *n*_1_ + *n*_2_) using *K* = 100 replications. **(A–F)** The analysis of data simulations in which 2, 5, 10, 25, 50, and 75% of bacterial features are not present in dataset **X**_2_ with respect to **X**_1_, respectively.

The same approach was used across different beta diversity metrics (Figure 4). The Jaccard diversity metric was the most sensitive and the weighted UniFrac was the least sensitive to observe the differences in the presence/absence between the datasets (Figures 4B–F). When more than 10% of ASVs were removed from dataset **X**_2_, no difference between datasets was observed by the UniFrac metric (Figure 4C), while with 25% removed, the BC and UF showed a comparable power and sample size (Figure 4D). In this simulated dataset, the weighted UniFrac distance needed the highest sample size to observe the difference (Figures 4D–F).

**FIGURE 5:**
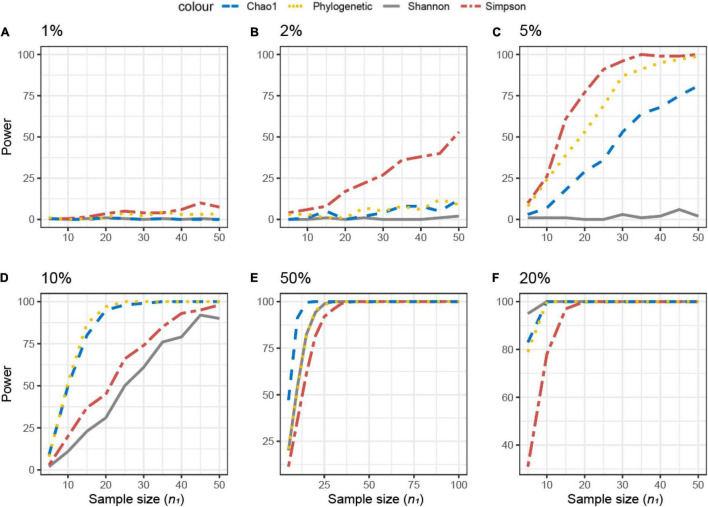
Empirical power of a two-sample Kruskal–Wallis test for the univariate comparison of two simulated microbiome datasets **X**_1_ and **X**_2_ [Simulated dataset 2: This simulation thus generates data under a differential abundant scenario where bacterial features are present in the different compositions in **X**_1_ and **X**_2_. The data have the same phylogenetic structure of the Human Microbiome Project (HMP) Gut data described in Table 1] using different alpha diversity metrics [phylogenetic, Chao1, Shannon’s, and Simpson’s indices; see Equations (3)–(5), and (6) in section “Materials and Methods”]. The empirical power is calculated using Equation (11) as a function of the sample size *n*_1_ of group 1 (with *n*_2_ = *n*_1_ and total sample size *n* = *n*_1_ + *n*_2_) using *K* = 1,000 replications. **(A–F)** The analysis of data simulations in which one-fourth of bacterial features in **X**_2_ are 1, 2, 5, 10, 15, and 20% more abundant than in **X**_1_, respectively.

The achievable power for Simulated dataset 2 is also shown as a function of the sample size for different percentages of differentially abundant ASVs (Figure 5). If ≤ 5% of the ASVs were differentially abundant in dataset **X**_2_ as compared with **X**_1_, Simpson’s metric needed the lowest sample size (is most sensitive) to observe differences between the data (Figures 5A–C). However, if 10% of the ASVs were differentially abundant, the PD and Chao1 were more sensitive and Simpson’s and Shannon’s metrics less sensitive (Figure 5D). With 15% of the ASVs differentially abundant, no differences were observed with Simpson’s metrics (Figure 5E).

**FIGURE 6:**
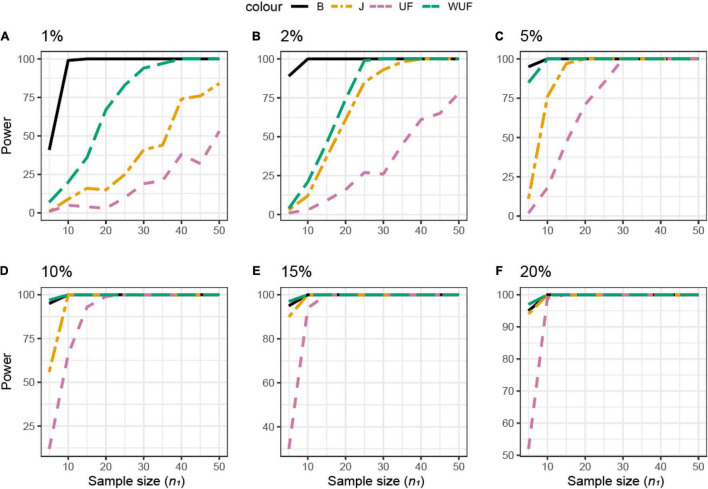
Empirical power of a permutational multivariate ANOVA (PERMANOVA) test for the multivariate comparison of two simulated microbiome datasets **X**_1_ and **X**_2_ [Simulated dataset 2: This simulation thus generates data under a differential abundant scenario where bacterial features are present in the different compositions in **X**_1_ and **X**_2_. The data have the same phylogenetic structure of the Human Microbiome Project (HMP) Gut data described in Table 1] using different beta diversity metrics [Bray–Curtis dissimilarity **(B)**, Jaccard (J), unweighted UniFrac (UF), and weighted UniFrac (WUF) distances; see Equations (7)–(9), and (6) in section “Materials and Methods”]. The empirical power is calculated using Equation (11) as a function of the sample size *n*_1_ of group 1 (with *n*_2_ = *n*_1_ and total sample size *n* = *n*_1_ + *n*_2_) using *K* = 100 replications. **(A–F)** The analysis of data simulations in which one-fourth of bacterial features in **X**_2_ are 1, 2, 5, 10, 15, and 20% more abundant than in **X**_1_, respectively.

The same approach was used across different beta diversity metrics (Figure 6). The BC distance was the most sensitive to observe differences, whereas UF needed the largest sample size. If 2% of the ASVs were differentially abundant, the power of the beta metrics was totally different; for example, a sample size of 15 would result in the power of 100 for the BC, 50 for weighted UniFrac, 40 for the Jaccard distance, and just 10 for UF (Figure 6B). However, if 10% of the ASVs were differentially abundant, all metrics would result in a power higher than 0.80 (Figures 6D–F).

### Power Analysis of Experimental Datasets: Chicken Dataset

**FIGURE 7:**
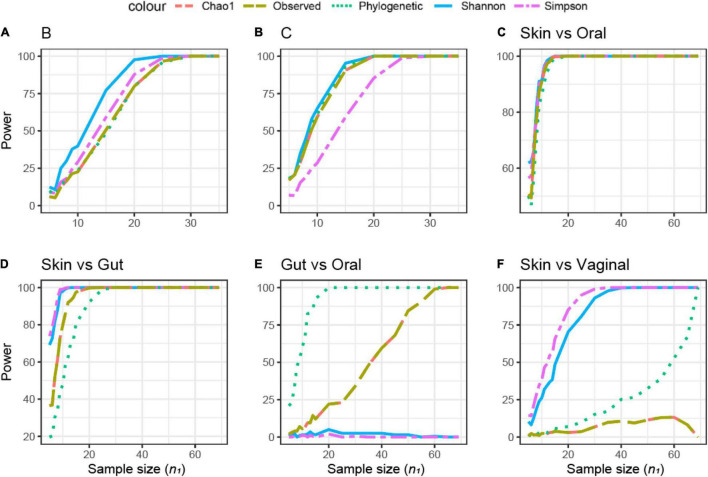
Empirical power of a two-sample Kruskal–Wallis test for the univariate comparison of two experimental microbiome datasets **X**_1_ and **X**_2_ from the Human Microbiome Projects (skin, gut, oral, and vaginal microbiome: dataset characteristics are given in Table 1). Differences between any two datasets **X**_1_ and **X**_2_ is assessed using different alpha diversity metrics [observed richness, phylogenetic diversity, Chao1, Shannon’s, and Simpson’s indices; see Equations (2)–(5), and (6) in section “Materials and Methods”]. The empirical power is calculated using Equation (11). Empirical power is calculated as a function of the sample size *n*_1_ of group 1 (with *n*_2_ = *n*_1_ and total sample size *n* = *n*_1_ + *n*_2_) using *K* = 1,000 replications (resampling)/tests for each sample size. **(A–F)** Different pairwise comparisons [A (poultry dataset B, feed A vs. B); B (poultry dataset C, feed A vs. B), skin microbiome vs. oral microbiome, skin vs. gut, gut vs. oral, and skin vs. vaginal].

**FIGURE 8:**
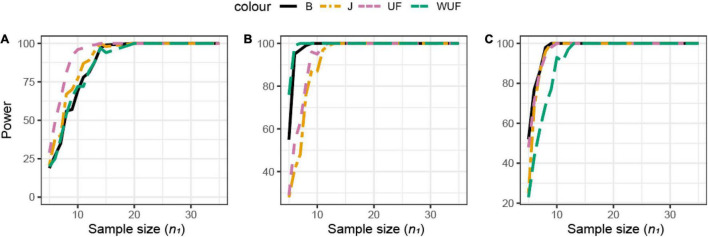
Empirical power of a two-sample Kruskal–Wallis test for the univariate comparison of two experimental microbiome datasets **X**_1_ and **X**_2_ from the three chicken datasets illustrated in Table 1. Differences between any two datasets **X**_1_ and **X**_2_ are assessed using different beta diversity metrics [Bray–Curtis dissimilarity **(B)**, Jaccard (J), unweighted UniFrac (UF), and weighted UniFrac (WUF) distances; see Equations (7)–(9) and (6) in section “Materials and Methods”]. The empirical power is calculated using Equation (11) as a function of the sample size *n*_1_ of group 1 (with *n*_2_ = *n*_1_ and total sample size *n* = *n*_1_ + *n*_2_) using *K* = 100 replications. **(A)** Analysis of chicken dataset A. **(B)** Analysis of chicken dataset B. **(C)** Analysis of chicken dataset C.

Shannon’s index was the most sensitive alpha metric and Chao1 and PD were the less sensitive metrics to observe a difference between the groups in dataset B (Figure 7A). In dataset B, Shannon’s index was also the most sensitive alpha metric, but Simpson’s index was the least sensitive metric (Figure 7B). UF distance was the most sensitive beta diversity metric to observe a difference between groups in dataset A (Figure 8A). The Jaccard distance was the only metric that showed that dataset C needed the smallest sample size, indicating that in dataset C, specific ASVs are differentially present between the groups (Figure 8). Weighted UniFrac was more sensitive than UF to observe a difference between the groups based on their microbial communities (Figure 8). In general, the alpha diversity measures were less sensitive to observe differences between the broilers than the beta diversity (Figures 7A,B, 8).

Although no difference in alpha diversity was observed between broilers fed with or without MCFAs raised in housing condition 1, the average daily gain and the average daily feed intake were lower in MCFA broilers (Kers et al., 2019). Therefore, the difference only observed based on the beta diversity might already be biologically relevant and hence sufficient to draw conclusions in this case. For this dataset, we observed that Shannon is the most sensitive alpha diversity metric to observe differences between groups, resulting in the lowest needed sample size. The sensitivity of the beta diversity, however, was different per dataset. Based on this retrospective power calculation, two conclusions can be drawn on this study design. First, not enough chickens were sampled to observe a difference in the alpha diversity between broilers fed with or without MCFA raised in dataset A. Second, 15 chicken samples instead of 35 samples per group would have resulted in the same conclusion.

### Power Analysis of Experimental Data: Human Microbiome Project Dataset

The samples in this dataset were collected from different body sites and are known to have a very distinct origin and therefore expected to be different in microbial composition. The comparison between different body sites showed a wide variation in sample size across different alpha diversities (Figures 7C–F). The difference in sample size was small in the comparison between skin and oral microbiome samples, a total of 10 samples (threshold power 80, 1-β) (Figure 7C). In the skin vs. gut microbiome samples, Simpson’s and Shannon’s alpha diversities did not differ, and the PD was the most sensitive to observe differences (Figure 7D). In contrast, when comparing the gut vs. the oral microbiome, the PD was the least sensitive to observe differences, whereas Shannon’s and Simpson’s metrics were different between the gut and oral samples (Figure 7E). In the skin vs. vaginal microbiome comparison, Simpson’s and Shannon’s alpha diversities were more sensitive than the observed/Chao1 and PD (Figure 7F). Based on the different beta diversity metrics, all comparisons between different body sites supported significant differences even when just five samples were compared (data not shown), due to the large difference between communities (Supplementary Figure 1). Therefore, the retrospective power calculations were not informative for this dataset.

### Are Microbiome Studies Underpowered?

**FIGURE 9:**
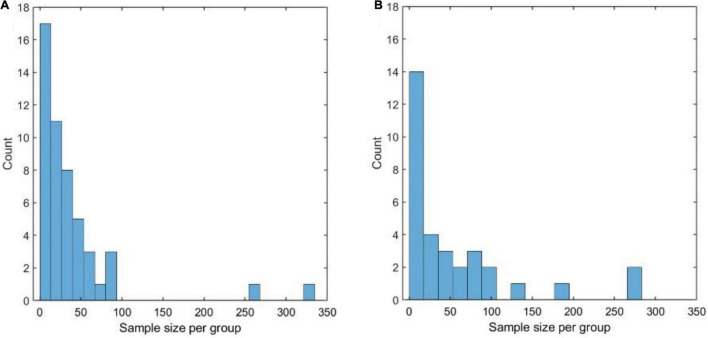
**(A)** Distribution of the sample size (per compared group) of the datasets that were analyzed, using the Chao1 diversity measure, in 28 of the 100 papers considered in the literature review. **(A,B)** Distribution of the sample size (per compared group) of the datasets that were analyzed, using permutational multivariate ANOVA (PERMANOVA), in 28 of the 100 papers considered in the literature review.

Figure 9A shows the distribution of the sample size (*n*_1_) of the datasets that were analyzed, using the Chao1 diversity measure in 28 of the 100 papers considered in the literature review. The distribution is highly skewed toward 0 with a median of 39 samples per group and a mode of 8 samples. Removing the two outlying studies with > 300 samples resulted in a median of 23 samples.

Even considering that Chao1 was one of the best-performing measures in both simulated and experimental datasets, these numbers appear to be worryingly low: on experimental data, which have a complicated structure that is impossible to replicate in simulations, it is rarely possible to attain a power of 80% with less than 40 samples per group. Similar considerations hold when PERMANOVA is applied (see Figure 9B), with a median group size of 22 and mode 3.

### Reporting of Power Analysis

One of the studies we examined in the literary review reported that sample size and power analysis were performed: “Sample sizes were chosen on the basis of pilot experiments and on our experience with similar experiments.” This is commendable, but we believe that the way forward is to employ and report in full a standardized summary of sample size calculations performed. The software G*power (Faul et al., 2007) generates a Protocol for power analysis. For instance, for a two-group comparison with a Mann–Whitney/Kruskal–Wallis test, a possible (modified) reporting is given in Table 4.

**TABLE 4:** Power analysis protocol for a Kruskal–Wallis test using Shannon’s alpha metric.

Power analysis protocol: univariate case—*alpha* diversity		
***t*-tests** – Means: Wilcoxon–Mann–Whitney test (two groups)		
**Options:**	A.R.E. method	
**Analysis:**	*A priori*: compute required sample size	
**Input:**	Tail(s)	= One
	Parent distribution	= Normal
	Effect size *d*	= 0.5
	Alpha metric	= Shannon
	α err prob	= 0.05
	Power (1 - β err prob)	= 0.8
	Allocation ratio N2/N1	= 1
**Output:**	Non-centrality parameter δ	= 2.51
	Critical *t*	= 1.66
	df	= 99.2
	Sample size group 1	= 53
	Sample size group 2	= 53
	Total sample size	= 106
	Actual power	= 0.803

This protocol is adapted from the protocol generated by the G*Power software (Faul et al., 2007).

Together with this, information should be provided on how effect size was determined, i.e., which pilot data were used and how the effect size was calculated.

A similar reporting protocol could be devised if simulations are used in a PERMANOVA setting (Table 5). Since simulation and/or pilot data must be used in this case, details on the simulations or pilot data should be reported. For instance, using Chicken data 1 as the pilot, one could report the following protocol, taking 100 resamplings of size 6 to calculate the achievable power:

**TABLE 5:** Power analysis protocol for a PERMANOVA test using the Bray–Curtis beta metric.

Power analysis protocol: multivariate case—*beta* diversity		
**Test –** PERMANOVA		
**Options:**	9,999 permutation	
	100 iterations	
**Analysis:**	Compute achievable power	
**Input:**	Beta metric	= Bray–Curtis
	α err prob	= 0.05
Number of groups		= 2
	Number of taxa	= 363
	Sample size group 1	= 6
	Sample size group 2	= 6
**Output:**		
Observed effect size (average) ϖ^2^		= 0.120682
	Min\Max effect size	= 0.025922\0.3500687
Observed effect size (average) *f*		= 0.2696886
Min\Max effect size		= 0.1319342\ 0.746349
	Numerator df	= 1
	Denominator df	= 10
	Power (1−β err prob)	= 0.97

*PERMANOVA, permutational multivariate ANOVA.*

## Discussion

The aim of this study was to assess how, and to what extent, different diversity metrics and compositions of the microbiota influence the needed sample size to observe statistically significant dissimilar groups. Based on our literature survey, we observed that Shannon’s and Bray–Curtis metrics are the most published metrics. This might be because they are often the most sensitive metrics to observe differences between groups, resulting in a lower sample size. Our results are in line with those of a previous literature that showed that the choice of distance metric may significantly influence the observed results (Koren et al., 2013).

A well-known phenomenon that can hamper progress in every research field concerns publication biases in reporting mainly positive findings (Ioannidis, 2005). In microbiota research, this might even occur rather unintentionally, by using certain alpha and beta diversity metrics, but it might also be that researchers selectively report only results for the metric that shows significance even when other metrics had been assessed during the analyses. Our results lead to the speculation that many microbiome studies may be underpowered or, conversely, only reporting evidence of very large effects that can be assessed to be statistically significant also with a small sample size. However, since effect size and test statistics are not reported, it is impossible to judge the quality of the results. This also hampers the use of published studies as pilot studies to perform power analysis and sample size calculations, as long as data are not *de novo* reanalyzed.

None of the 100 microbiome studies that we have considered reported the effect size. A collaborative project aiming to investigate the reproducibility of 100 high-profile psychological studies reported that the average effect size observed in the replication studies was approximately half the magnitude of those given in the original studies, leading to a replication success of only 36% (Open Science Collaboration, 2015). The lack of reported effects makes it impossible to analyze retrospectively microbiome studies and to perform a meta-analysis and, more importantly, makes it impossible to check the consistency of the statistical analysis or detect errors.

On the basis of this, reporting of effects and test statistics should be made compulsory in microbiome studies. For the highly used Kruskal–Wallis test, the *H* test statistic is given by (in absence of ties)


(12) $$H=\frac{12}{N\left(N-1\right)}\sum_{i}n_{i}R_{i}^{2}-3N+1$$

where *N* is the total number of samples, *n*_*i*_ is the number of samples in group *i*, and *R*_*i*_ is the average rank of observations in the *i*th group. Note that *H* (or alternative formulas) obtainable from most software packages.

For the Kruskal–Wallis, the most common effect is the η^2^, which is defined as


(13) $$\eta^{2}=\frac{H-k+1}{N-k}$$

where *H* is the value obtained in the Kruskal–Wallis test and *k* is the number of groups. For instance, for the comparison of the two feedings (Feed A and B; see Table 1) from the chicken dataset using the observed alpha diversity, one could report the following:

Feed A (*n*_1_ = 35) and Feed B (*n*_2_ = 35) samples were compared with Kruskal–Wallis test using the Chao1 metric: H (*df*) = H (1) = 14.68, *p*-value 0.0001, η^2^ = 0.66, δ = 0.58,

where *df* indicates the degrees of freedom.

Note here that for a two-group comparison, the Kruskal–Wallis test is equivalent to the Wilcoxon–Mann–Whitney (WMW) test (Hoffman, 2015; Happ et al., 2019). For the WMW test, Cohen’s δ effect size definition (Equation 1) also applies (Lakens, 2013). This greatly simplifies power analysis and sample size calculations: we advise to also report δ when two groups are considered.

In addition, performing power analysis for a Kruskal–Wallis is not a simple matter and requires the use of a rather advanced statistical machinery (Fan et al., 2011; Fan and Zhang, 2012); for instance, such calculations are not included in G*power (Faul et al., 2007), which is the most complete software for power analysis. The Kruskal–Wallis is the nonparametric counterpart of one-way ANOVA and as such is used in situations where there are more than two groups. However, whereas power analysis and sample size calculation for a one-way ANOVA with more than two groups are “easily” accessible within R or other software packages, this is not the case in the Kruskal–Wallis testing. We could locate an R package “MultNonParam” (Kolassa and Jankowski, 2020) that performs power analysis for the Kruskal–Wallis test with more than two groups; however, it requires the specification of the offsets for the various populations, under the alternative hypothesis. Relating such tools to determine the effect size observed in microbiome data is a matter we believe to be worthy of exploration and brings us back to the problem that statistics and effect size are not easily available for microbiome studies.

The principle of reporting the effect size should also apply when testing is performed using beta diversity metrics, in which case the PERMANOVA pseudo *F*-statistics (see Equation 10) and the effect size should be reported. Typical effect measures in ANOVA are Choen’s *f*^2^, η^2^, and ϖ^2^:

Feed A (*n*_1_ = 35) and Feed B (*n*_2_ = 35) samples were compared with PERMANOVA test using the Unweighted Unifrac metric: *F*(df_*B*_, df_*W*_) = *F*(1, 68) = 6.27, *p*-value = 0.0001, *f*^2^ = 0.092, 1,000 permutations,

where *df*_*B*_ and *df*_*W*_ indicate the between-groups and within-groups degrees of freedom, respectively. These notations follow the guidelines of the American Psychological Association, which provides standardized formats for the reporting of statistical analysis for statistical procedures (American Psychological Association, 1994).

For PERMANOVA, the matter complicates considerably: to estimate statistical power and calculate sample size, one must quantify the expected within-group variance and the effect to be expected when comparing two or more groups. A package like micropower (Kelly et al., 2015) in principle allows estimation of PERMANOVA effects quantified by the ϖ^2^ value (limited to the UniFrac measure): unfortunately, to the best of our knowledge, the package seems not to be maintained and lacks a proper manual. The original paper presents a table with effects calculated from different studies that could be used as a guide; however, this metric is not standard. It can be calculated from the PERMANOVA table as


(14) $$\omega^{2}=\frac{SS_{effect}-df_{effect}MS_{residual}}{SS_{effect}+MS_{residual}}$$

For the chicken data, we observed ϖ^2^ values in the range 0.04–0.1, depending on the beta metrics, and these are consistent with those reported in Table 1 from Kelly et al. (2015).

A more common effect measure is Cohen’s *f*^2^-value; i.e., the between-group to within-group ratio can be easily obtained by the ANOVA table provided by software like the R package Vegan by taking the ratio between the Treatment sum of squares and the Residual sum of squares. The *f*^2^-value is used for power calculation in the ANOVA setting; however, it should not be used to perform sample size calculation for PERMANOVA, not even to obtain a rough indication, since the corresponding *F*-statistics do not follow an *F* distribution. For instance, when comparing Feed A and B from the first chicken data with PERMANOVA, we can derive a Cohen’s *f*^2^ = 0.38; if this value is used to perform power calculation for an ANOVA with two groups with power 80% at α = 0.01, we obtain that 42 samples per group are needed. However, comparing the results in Figure 8, we see that a 100% power can be obtained with 25 samples per group, regardless of which measure is used: this is a clear indication that power analysis for PERMANOVA can be obtained only by means of simulations. In this light, Figure 8 can be viewed as *a priori* power calculations using pilot data.

Furthermore, there is a convention, more or less widely accepted, of classifying Cohen’s δ effect, which we have used in the power calculation for the Kruskal–Wallis, into trivial (δ < 0.2), small (δ = 0.2), medium (δ = 0.5), and large (δ > 0.8). However, this classification is based on what is observed in psychology and does not apply automatically to other fields of research (Saccenti and Timmerman, 2016). In microbiome studies, the effects may be in the same order of magnitude that may be considered large or very large using the standard convention.

We have seen that different alpha and beta diversity metrics lead to different study power: on the basis of this observation, one could be naturally tempted to try all possible metrics until one or more are found that give a statistically significant test result, i.e., *p*-value < α. This way of proceeding is one of the many forms of the so-called *p*-value hacking (*p*-hacking) (Simmons et al., 2011). *p*-Hacking (also called data dredging, significance chasing, significance questing, or selective inference (Wasserstein and Lazar, 2016)) is the improper use of data (like adding or removing observations) or statistical procedures (like applying many different tests) until a configuration is found that produces a statistically significant result at the desired confidence level (Smith and Ebrahim, 2002). *p*-Hacking is an illegitimate practice that promotes unreproducible results, polluting literature and adding to publication bias (Ioannidis, 2005; Jager and Leek, 2014; Raj et al., 2018).

## Conclusion

To this end, in our opinion, the only way to protect ourselves from (the temptation of) *p*-hacking would be to *publish*, and we stress here the word publish, a statistical plan before experiments are initiated: this practice is customary for clinical trials where a statistical plan describing the endpoints and the corresponding statistical analyses must be disclosed before the start of the study and must be adhered to if results are going to be published (Gamble et al., 2017). This is the only guarantee that data analysis is not manipulated toward artificially inflated significant results. We appreciate that clinical trials are inherently different from microbiome (and other omics) studies, which are often exploratory in nature, but as far as statistics are concerned, they are the prey of the same traps and pitfalls. It is obvious that such a change in the approach to microbiome studies requires the concerted cooperation of researchers, journal editors, reviewers, and publishers.

## Data Availability Statement

HMP data used in this study are available at https://github.com/mibwurrepo/Microbial-bioinformatics-introductory-course-Material-2018/tree/master/input_data; http://doi.org/10.5281/zenodo.1436630 (Shetty et al., 2020). The poultry data are available at https://www.ncbi.nlm.nih.gov/bioproject/ with accession number PRJNA553870. Simulated datasets are available at https://github.com/mibwurrepo/KersSaccenti-Power.

## Author Contributions

JK and ES wrote the manuscript. Both authors read and approved the final manuscript.

## Conflict of Interest

The authors declare that the research was conducted in the absence of any commercial or financial relationships that could be construed as a potential conflict of interest.

## Publisher’s Note

All claims expressed in this article are solely those of the authors and do not necessarily represent those of their affiliated organizations, or those of the publisher, the editors and the reviewers. Any product that may be evaluated in this article, or claim that may be made by its manufacturer, is not guaranteed or endorsed by the publisher.

## Footnotes

1
https://pubmed.ncbi.nlm.nih.gov/

## Abbreviations

Anosim: analysis of similarities
Asv: amplicon sequence variant
Bc: Bray–Curtis index
Hmp: Human Microbiome Project
J: Jaccard distance
Mcfa: medium-chain fatty acids
Otu: operational taxonomic unit
Permanova: permutational multivariate Anova
Pd: phylogenetic diversity
Uf: UniFrac.
