# Supplementary material for: The Power of Microbiome Studies: Some Considerations on Which Alpha and Beta Metrics to Use and How to Report Results
Source: Front Microbiol. 2022 Mar 3;12:796025. doi: 10.3389/fmicb.2021.796025 (PMC8928147; doi:10.3389/fmicb.2021.796025)
Supplement: Supplementary file 1 [file Data_Sheet_1.docx]

# Supplementary figure


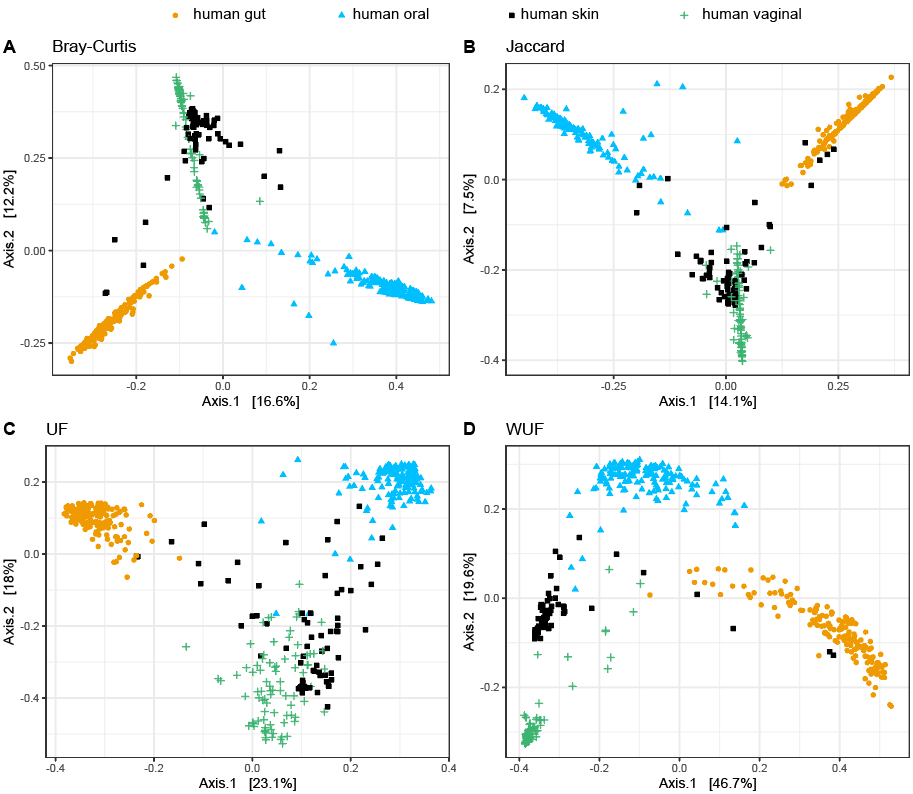


**Figure S1**

Principal coordinate plots (PCoA) based on (a) Bray-Curtis and (b) Jaccard and (c) unweighted UniFrac and (d) Weighted UniFrac distances between the different human sample types. Different colours indicate different sample types.
